# Using clinical audit to improve the quality of obstetric care at the Tibetan Delek Hospital in North India: a longitudinal study

**DOI:** 10.1186/1742-4755-3-4

**Published:** 2006-06-07

**Authors:** Stewart W Mercer, Katherine Sevar, Tsetan D Sadutshan

**Affiliations:** 1 Section of General Practice and Primary Care, Division of Community-based Sciences, University of Glasgow, UK; 2 General Surgery, Queen Margaret Hospital, Dunfermline, UK; 3 Tibetan Delek Hospital, Gangchen Kyishong, Dharamsala, Himachal Pradesh, India

## Abstract

**Background:**

The Tibetan Delek Hospital is a small general hospital providing primary and secondary care for the Tibetan refugee community and the local Indian population in Dharamsala, Himachal Pradesh, North India. In a baseline clinical audit of intrapartum care at the Tibetan Delek Hospital in 1996, high levels of postpartum haemorrhage associated with poor medical management of the third stage of labour, plus inappropriate transfer of women in labour were observed. These audit findings prompted the implementation of changes in the delivery of intrapartum care and follow-up audit cycles to monitor the ongoing effect of these changes.

**Methods:**

The delivery of intrapartum care was modified in two ways. Firstly, nurses, midwives, and doctors were re-trained in the active management of the third stage of labour, which involved the administration of intramuscular syntocinon plus ergometrine with delivery of the anterior shoulder. Secondly partograms were introduced to help rationalise the management of labour, and in particular decisions about when to transfer women in labour. Follow up audits were conducted in 1997, 1998, and 2003 to quantify the effects of these changes. The key measures for improvement included the documented incidence of postpartum haemorrhage and the number of women transferred inappropriately for failure to progress in labour.

**Results:**

A sustained reduction of approximately 50% in the incidence of postpartum haemorrhage was observed after the introduction of active management of the third stage of labour. The introduction of the routine use of partograms was associated with a more rational decision-making process regarding transfer during labour.

**Conclusion:**

Introducing and maintaining a clinical audit cycle can lead to improvements in the quality of obstetric care in a refugee population.

## Background

Increasing the effectiveness and efficiency of health services is important everywhere but particularly so in developing countries with limited resources [1]. In the UK, clinical audit is seen as a useful, but not always effective tool in quality improvement. There are remarkably few reports on the benefits of clinical audit in the developing world, though audit has been used in maternity care as part of the Safe Motherhood Initiative [2]. Effective change in the developing world may have important lessons for developed nations [3]. The present study reports on the benefits of clinical audit in reducing complications in labour in the Tibetan Delek Hospital, Dharamsala, Himachal Pradesh, North India, which is a general hospital providing primary and secondary care for the Tibetan refugee community and the local Indian population.

Maternal mortality is the biggest threat to a woman's health in the developing world with post-partum haemorrhage (PPH) as one of the most common contributing factors [4,5]. The primary cause of PPH is uterine atony which accounts for 70% of cases [4]. Reducing the incidence of PPH can lead to a reduction in maternal mortality in the developing and developed world [4,5].

Partogram use has become routine in the management of labour in many countries around the world, and can be helpful in the management and decision making process [6-8].

Both the incidence of postpartum haemorrhage and the appropriateness of transfers of women in labour were considered to be causes for concern after the baseline audit in 1996 (see below and table). There are no on-site obstetricians or surgical facilities at the Delek Hospital so those women experiencing prolonged or difficult labours, or those potentially requiring Caesarean section are transferred to obstetricians at the nearest district or private hospital (a journey of 20–40 min by jeep, often followed by lengthy delays on arrival). The 1996 baseline audit suggested that decision-making by staff regarding transfer was often inappropriate and not based on recorded details of progression of labour. A response to this was the introduction of the routine use of the WHO partogram [6,7], with the aim of quantifying events occurring during labour and qualifying decisions taken regarding transfer.

## Methods

The case-notes of all labour admissions in 1996 were reviewed in January 1997 and baseline figures recorded for the number of admissions in labour; the number transferred, – including the proportion of uncomplicated pregnancies in primiparous women that were transferred for 'failing to progress', the number of augmentations in labour; and the incidence of complications, including PPH. Only blood loss recorded in the notes as 500 mls or more was defined as PPH and only those cases were included. Follow-up audits were conducted one year later (all admissions in 1997), two years later (all admissions in 1998) and seven years later (all admissions in 2003). In the follow-up audits, the number of women in labour assessed by using the partogram was noted, and the quality of the information recorded on the partogram was coded by the clinical researchers as follows: 'complete' if all the required information had been recorded on the partogram, 'incomplete but helpful' if some of the information was not recorded but the partogram was of potential clinical usefulness, and 'incomplete and unhelpful' if there was information missing that rendered the partogram unhelpful or potentially misleading.

Feedback was delivered to the staff at each time point by giving seminars to all the clinical staff after each audit cycle, by having one-to-one question and answer sessions with the nursing staff before and after each audit cycle, and by publishing the audits each year in the hospital newsletter.

As this was a clinical audit of routine care, ethical approval was not considered to be necessary. There was no ethical board in the Tibetan-Government-in exile in Dharamsala in place at the time.

## Results

The baseline audit revealed apparently high rates of post-partum haemorrhage (PPH) (table). Discussion with the nursing and medical staff revealed that although the policy of the hospital was 'active management of the third stage of labour '[5], routine practice at this time was either not to give any oxytocin or to give intramuscular Ergometrine 0.2 mg (1 vial) *after *the delivery of the placenta. This reflected a concern that to give it before the third stage might lead to retained placenta. The baseline audit also revealed that almost 1 in 5 labour admissions were transferred before delivery to obstetricians at the nearest district or private hospital. Seventy-five percent (8/11) of these transfers were for supposed "failure to progress" in primiparous women with no other complications. The details of the progression of labour from time of admission to transfer were poorly documented, and it was clear that the decision-making process regarding transfer was somewhat haphazard.

The findings of the baseline 1996 audit were discussed with the medical and nursing staff, both on a group basis and on a more informal individual basis. Two key changes were agreed upon and introduced: the routine use of a partogram and the active management of the third stage of labour with intramuscular '*Syntrometrine*' (5U Syntocinon and 0.5 mg Ergometrine) as the anterior shoulder is delivered [5]. Staff were trained in the use of the partogram; how to fill it in correctly and how to recognise 'failure to progress'.

The effects of the changes introduced can be seen from the audit data at each time point (table 1). Follow-up at 1 year (1997) revealed the uptake of using the partogram was good but the quality and accuracy of the information recorded was poor with almost a third of partograms being completed in a way which was unhelpful and potentially misleading. However, follow-up at 2 years (1998), after a second round of feedback and discussion, showed a substantial improvement with 90% of the partograms being completed accurately enough to be clinically helpful in decision-making. In conjunction with the uptake of partogram use, the percentage of women being transferred fell substantially, with fewer transfers for "failure to progress" in uncomplicated pregnancies in primiparous women. The follow-up at seven years (2003) revealed a sustained reduction in the percentage of women being transferred. Partograms were still being used routinely, although there was some decrease in the quality of the information recorded.

**Table 1:** Management of labour at Delek Hospital before and after introduction of partogram and active management of the third stage

	Baseline Audit; 1996	Follow up Audit; 1997	Follow up Audit; 1998	Follow up Audit 2003
Number of admissions in labour	70	110	65	90
Number of women transferred (%)	11 (19%)	15 (14%)	6 (9%)	6 (7%)
Reason for transfer 'Failure to Progress' in uncomplicated primiparous women; n (%)	8 (75%)	3 (20%)	0 (0%)	1 (17%)
Partograms used at Delek n (%)	0 (0%)	88 (80%)	57 (87%)	77 (86%)
Partogram information n (%);				
- complete and accurate	-	9 (10%)	34 (60%)	13 (17%)
- incomplete but helpful	-	53 (60%)	17 (30%)	52 (68%)
- incomplete and unhelpful	-	26 (30%)	6 (10%)	12 (15%)
Augmentation in labour at Delek n (%)	0 (0%)	1 (1%)	7 (11%)	6 (7%)
Complications at Delek n (%);				
- PPH	7 (12%)	6 (6%)	3 (5%)	5 (6%)
- Retained placenta	3 (5%)	2 (2%)	0 (0%)	0 (0%)

The follow-up audits in 1997, 1998 and 2003 also showed marked reductions in the documented incidence of both PPH and retained placenta compared with the baseline data at 1996 (table 1). This apparent halving of the incidence of PPH with active management is consistent with the literature from large randomised controlled trials [5].

## Discussion

Clinical audit of obstetric care at the Tibetan Delek Hospital, North India, resulted in the introduction of two simple changes in management, namely the introduction of partograms and the active management of the third stage of labour. Completing the audit cycle has indicated that these changes were incorporated successfully into routine care, apparently with considerable clinical benefit. However, there has been some decrease in the quality of the information recorded on the partograms, and our next step will be to re-train the staff in the use of the partogram. Since the initial training and feedback was introduced there have been many changes in the nursing and medical personnel, including a change of matron and deputy matron, one Tibetan doctor who left the service and three new Tibetan doctors who joined after the audit was started, and these changes may account for the decrease in the quality of partogram recording.

Whilst a clear benefit has been elicited from the introduction of these interventions there can be potential problems with 'external audit'. In this case the audit was initiated and carried out by the first author (SWM) when working as a volunteer non-tibetan doctor at the hospital, and followed up in 2003 by the second author (KS) who was also a foreigner. Staff at the Tibetan Delek Hospital remains enthusiastic about the changes put in place, and the full involvement, strong commitment and team-work has been crucial to achieving this aim. Once the changes were incorporated into routine care, the staff themselves embraced them as they immediately saw the benefits. Feeding back the audit data to show the improvements helped to further imbed the changes, and alleviate concern, for example about increasing the risk of retained placenta. It will be important, however to maintain the audit cycle and we feel it is now essential that this task is taken on by a designated, permanent staff member with proper training, supervision, and protected time.

## Conclusion

The introduction of a clinical audit cycle from 1996–2003 at the Delek Tibetan Hospital in North India has been associated with successful changes in the management of labour and improved outcomes, including a 50% reduction in PPH. Clinical audit may have an important role to play in improving quality of medical care in the developing world.

## Competing interests

The author(s) declare that they have no competing interests.

## Authors' contributions

Dr SW Mercer is a GP and Senior Clinical Research Fellow at Glasgow University.. He suggested and conducted the audit from 1997–1999, and supervised the follow up audit in 2003.

Dr Katherine Sevar conducted the follow-up audit in 2003, and helped in the drafting of the manuscript.

Dr Tsetan Sadhutshang is the Chief Medical Officer at the Tibetan Delek Hospital, and hospital administrator. He supported the audit and organized the feedback meetings with the nursing and medical staff.

## Funding

The study was unfounded. SWM received a Travel Award from the RCGP, UK in 1999 to complete the follow up of the audit.

## References

[B1] Smits HL, Leatherman S, Berwick DM (2002). Quality improvement in the developing world. Int J Qual Health Care.

[B2] Hussein J, Goodburn EA, Damisoni H, Lema V, Graham W Monitoring obstetric services; putting the 'UN Guidelines' into practice in Malawi: 3 years on. International Journal of Gynaecology and Obstetrics.

[B3] Berwick DM (2004). Lessons from developing nations on improving health care. Br Med J.

[B4] Kwast BE (1991). Postpartum haemorrhage: its contribution to maternal mortality. Midwifery.

[B5] Elbourne DR, Prendiville WJ, Carroli G, Wood J, McDonald S (2004). Prophylactic use of oxytocin in the third stage of labour (Cochrane Review). The Cochrane Library.

[B6] Lavender T, O'Brien P, Hart A (2006). Effect of partogram use on outcomes for women in spontaneous labour at term. Cochrane Systematic Reviews.

[B7] Walraven GE (1994). WHO partogram. Lancet.

[B8] Dujardin B, De Schampheleire !, Sene H, Ndiaye F (1992). Value of alert and action lines on the partogram. Lancet.

